# Genome-wide identification and characterization of transfer RNA-derived small RNAs in *Plasmodium falciparum*

**DOI:** 10.1186/s13071-019-3301-6

**Published:** 2019-01-15

**Authors:** Zhensheng Wang, Chunyan Wei, Xiao Hao, Weiwei Deng, Lianhui Zhang, Zenglei Wang, Heng Wang

**Affiliations:** 1 Department of Microbiology and Parasitology, Institute of Basic Medical Sciences Chinese Academy of Medical Sciences, School of Basic Medicine Peking Union Medical College, 5# Dong Dan San Tiao, Beijing, 100005 People’s Republic of China; 2 NHC Key Laboratory of Systems Biology of Pathogens, Institute of Pathogen Biology, Chinese Academy of Medical Sciences and Peking Union Medical College, Beijing, People’s Republic of China

**Keywords:** *Plasmodium falciparum*, Transfer RNA, Short RNAs, Small RNAs, tRFs

## Abstract

**Background:**

Transfer RNA (tRNA)-derived fragments (tRFs) have been widely identified in nature, functioning in diverse biological and pathological situations. Yet, the presence of these small RNAs in *Plasmodium* spp. remains unknown. Systematic identification and characterization of tRFs is therefore highly needed to understand further their roles in *Plasmodium* parasites, particularly in the virulent *Plasmodium falciparum* parasite.

**Results:**

Genome-wide small RNAs with sizes ranging from 18–30 nucleotides from *P. falciparum* were deep-sequenced *via* Illumina HiSeq 2000 technology. In-depth analysis revealed the presence of a vast number of small RNAs originating from tRNA-coding genes, responsible for 22.4% of the total reads as the second predominant group. Three *P. falciparum*-derived tRF types (ptRFs) were identified as 5'ptRFs, mid-ptRFs and 3'ptRFs. The majority (90%) of ptRFs were derived from tRNAs that coded eight amino acids: Pro, Phe, Asn, Gly, Cys, Gln, His and Ala. Stem-loop reverse transcription polymerase chain reaction further confirmed the presence of tRFs in the blood stages of *P. falciparum*. Four new motifs with an enriched G/C feature were determined at cleavage sites that might guide the generation of ptRFs.

**Conclusions:**

To our knowledge, this is the first report of a genome-wide investigation of ptRFs from *Plasmodium* species. The identification of ptRFs reveals a complex small RNA system manipulated by the malaria parasite, and might promote research on the function of tRFs in the pathogenesis of *Plasmodium* infections.

**Electronic supplementary material:**

The online version of this article (10.1186/s13071-019-3301-6) contains supplementary material, which is available to authorized users.

## Background

A growing amount of data suggests that small RNAs play critical regulatory roles in many biological processes in both prokaryotes [1–3] and eukaryotes [4, 5]. Other than the three major types of small RNAs including microRNAs (miRNAs), endogenous small interference RNAs (endo-siRNAs) and PiWi-interacting RNAs (piRNAs) [6, 7], the advance in deep sequencing technology has unveiled new classes of small RNAs with novel features and functions. One such class is those derived from transfer RNAs (tRNAs), commonly referred to as tRFs (tRNA-derived RNA fragments), which were once mis-annotated as miRNAs [8, 9].

tRFs are typically short in length, ranging from 12–30 nucleotides (nt) [10]. Despite the fact that the nomenclature used to refer to the subclass of tRFs varies in the literature, they are generally classified in terms of their origin: 5'tRFs are generated from 5' end cleavage in the D-arm of mature tRNAs, 3'tRFs or 3'CCA tRFs are produced from 3' end processing of mature tRNAs in the TΨC-arm and contain a CCA post-transcriptional modification, 3'UtRFs or tRF-1 are derived from the 3' end of a pre-tRNA, and endogenous tRFs (itRFs or mid-tRFs) originate from a combination of cleavage from the anticodon loop domain and either D-arm or TΨC-arm of mature tRNAs. Single cleavage occurring in the anticodon loop could lead to generation of a conventional class of tRNA-derived RNAs, namely tRNA-halves (~ 30–50 nt). Apart from the yield of tRNA-halves from mature tRNA under stress conditions, the biogenesis of tRFs as well as their functions remain to be fully understood.

With increased research interest in tRFs, mounting evidence has linked the production of tRFs to cancer [11], neurodegenerative disorders, viral infection and other pathological conditions. They have been involved in cellular proliferation, RNA interference pathway and regulate gene translation at the post-transcriptional level, similar to miRNAs (reviewed in [10, 12]). Moreover, the interactions in many cases between tRFs with the functional proteins in miRNA pathway such as argonaute (AGO) protein families [13, 14], PIWI [15] and DICER proteins [16] further imply the possibility of tRFs behaving like miRNAs and siRNAs.

Malaria remains the most grievous protozoan load due to its severe prevalence and lethality in the world, particularly in Southeast Asia and Africa. More than 400,000 deaths were recorded in 2017 by the WHO Malaria Report [17]. Of the five human malaria parasite species, *Plasmodium falciparum* is the most virulent, causing the majority of malaria-related mortality. In *P. falciparum*, as well as in other *Plasmodium* parasites, active RNA interference apparatus [18] and canonical miRNA molecules were found to be absent [19, 20], as confirmed by computational and experimental evidence. The lack of DICER and AGO homologs in *Plasmodium* parasites suggests that these organisms might use an alternative pathway to accomplish the intense post-transcriptional regulation needed during the rapid morphological change in the intraerythrocytic cycle. It is possible that small RNAs such as tRFs might be involved. Until now, the existence and characterization of tRFs in *Plasmodium* parasites has not been evaluated. tRFs have been identified in several protozoan organisms including *Tetrahymena* [21], *Giardia lamblia* [22], *Trypanosoma cruzi* [23] and the exosomes from *Leishmania donovani* [24]. This provides credence that these deeply conserved molecules are expressed in protozoan species, even though their classifications and functions remain unclear. With this inspiration, we sought to investigate tRFs in the malaria parasite, in which the machinery associated with small RNA biogenesis is thought to be either entirely lost or extensively simplified [18–20].

Here, we focused on the deadly *P. falciparum* and took the advantage of deep sequencing technology to comprehensively analyze genome-wide high-throughput sequencing data from *P. falciparum*-derived small RNAs. To our knowledge, we report for the first time the expression of novel, endogenous *Plasmodium* tRFs (ptRFs) and their global distribution and characterization in blood stages of *P. falciparum*.

## Methods

### Maintenance of *P. falciparum*

The standard *P. falciparum* laboratory strain 3D7 (Pf3D7) was obtained from ATCC (Manassas, VA, USA) and maintained within fresh human red blood cells (RBCs) at 5% hematocrit in RPMI-1640 medium supplemented with 0.5% (w/v) albumax II (Invitrogen, Carlsbad, CA, USA), as previously described [25]. Parasitemia was monitored by Giemsa staining of blood smears.

### Small RNA purification and high-throughput sequencing

The mixed-stage infected RBCs were lysed with 0.05% saponin and washed three times with RPMI-1640 to collect parasites. Total RNA from the parasite pellet was extracted using Trizol reagent (Invitrogen) following the manufacturer’s instructions and was quantified by absorbance at 260 nm. RNA was then separated by urea-denatured 15% polyacrylamide gel electrophoresis, and bands of small RNAs of 18–30 nt in length were extracted and purified. Small RNA library construction and sequencing was performed following commercial protocols. Briefly, the small RNA molecules were ligated to 5' and 3' adaptors sequentially and then converted to cDNA with reverse transcription followed by polymerase chain reaction (PCR) amplifications. Approximately 20 μg of reverse transcription (RT)-PCR products per sample were sequenced using an Illumina Hiseq 2000 platform (Illumina, San Diego, CA, USA) by Beijing Genomics Institute (BGI, Shenzhen, China).

### Small RNA data analysis

A total of 14,828,877 reads were initially obtained from the sequencing of small RNA libraries. Raw sequences were pre-cleaned to remove low-quality reads and 10,230,166 high-quality reads were then trimmed to discard the 3' and 5' adaptors, sequences containing the polyA tail, and those smaller than 18 nt or larger than 30 nt. This dataset was mapped to the genome of the *P. falciparum* 3D7 strain (PlasmoDB, release 36, http://plasmodb.org/plasmo/) using BLASTN to allow the removal of any contamination from human RNA segments to yield a clean data pool. These data were analyzed in both total and unique aspects. Total reads, also referred to as redundant reads in the literature, indicated multiple reads aligned around the same location, representing high abundance of the RNA. To demonstrate the diversity of the small RNA, the total reads were collapsed to remove the redundant ones, resulting in single consensus aligned unique reads. The clean-read counts were normalized as a relative number per one million reads (RPM) in both total and unique categories for further analysis. Information of the 45 *P. falciparum* tRNA genes was downloaded from the PlasmoDB database. By performing BLAST alignments, data from the mapped *Plasmodium* tRNA-derived segments were extracted and further analyzed. The expression intensity of the ptRFs against the whole genome was sequentially calculated with the “Build” function of Bowtie2 (v.2.1.0.0) and the pipeline of TopHat (v.2.0.9) and Cufflinks (v.2.2.1). The data extraction of each type ptRFs was performed by compiled Perl (v.5.22) codes. The statistical analysis for the Pearsonʼs correlation coefficients was performed using the “cor” function in R package *stats*. The plots were drawn mainly with the *ggplot2* package in R language (v.3.5.0).

### Motif analysis

Gapped Local Alignment of Motifs (GLAM2 v1056) [26] and Motif Comparison Tool (Tomtom v.4.12.0) [27] were used to predict and compare the motifs around the cleavage sites of the tRFs. For the GLAM2 algorithm, the adopted parameters for motif predication were set as default values, except that only the given strand was aligned. The outputs were directly submitted into the Tomtom algorithm for comparing the predicted motifs against the known RNA motif database. All known motifs were selected to be calculated using an e-value of < 10 followed by the adoption of the function for Pearsonʼs correlation coefficient.

### Stem-loop RT-PCR

Total RNA templates were reverse-transcribed into cDNAs using the EasyScript One-Step gDNA Removal and cDNA Synthesis SuperMix Kit (TransGen Biotech, Beijing, China) according to the manufacturer’s instructions. Specific stem-loop reverse transcription primers (Additional file 1: Table S1) were designed based on the sequence of each ptRFs as described previously [28, 29]. The PCR cycling conditions were as follows: initial denaturation at 94 °C for 5 min, followed by 30 cycles of denaturation at 94 °C for 5 s, annealing at 50 °C for 15 s and extension at 72 °C for 15 s, with a final extension at 72 °C for 5 min. The PCR products were separated on 12% polyacrylamide gel. The expected sizes of the PCR products were calculated by the length of each ptRF plus 40 base pairs of nucleotides technically introduced in the design of specific stem-loop primers. Negative controls, absent with either DNase I-treated RNA or reverse transcriptase in RT reactions, were performed to verify the accuracy and specificity of the stem-loop RT-PCR. The sequences of RT-PCR primers are listed in Additional file 1: Table S2.

## Results

### General features of tRNA-derived small RNAs in *P. falciparum*

The quality control strategies gave rise to a total of 9,767,982 high-quality clean reads ranging from 18 to 30 nt in length. Of these, 75.7%, equivalent to 7,392,533 reads, were perfectly mapped to the nuclear genome, whereas 0.09 and 1.72% matched to the apicoplast and mitochondrial sequences, respectively (Table 1). Sequences without genomic match were assumed to be host-derived small RNAs, which might be erythrocytic miRNAs translocated into the parasite cytosol as previously reported [30].

**Table 1:** Classification of small RNAs mapping to the *P. falciparum* genome

Categories	Total	Unique
Clean reads	9,767,982	957,443
Unmapped reads	2,198,260	312,761
Nuclear genome	7,392,533	635,476
mRNAs	634,793	422,374
rRNAs	4,464,246	130,442
tRNAs	2,184,619	28,091
snRNAs/snoRNAs	110,769	8,287
Annoted ncRNAs	95,718	7107
Unannoted small RNAs	80,444	39,175
Apicoplast	9298	2415
Mitochondria	167,891	6791

**Fig. 1:**
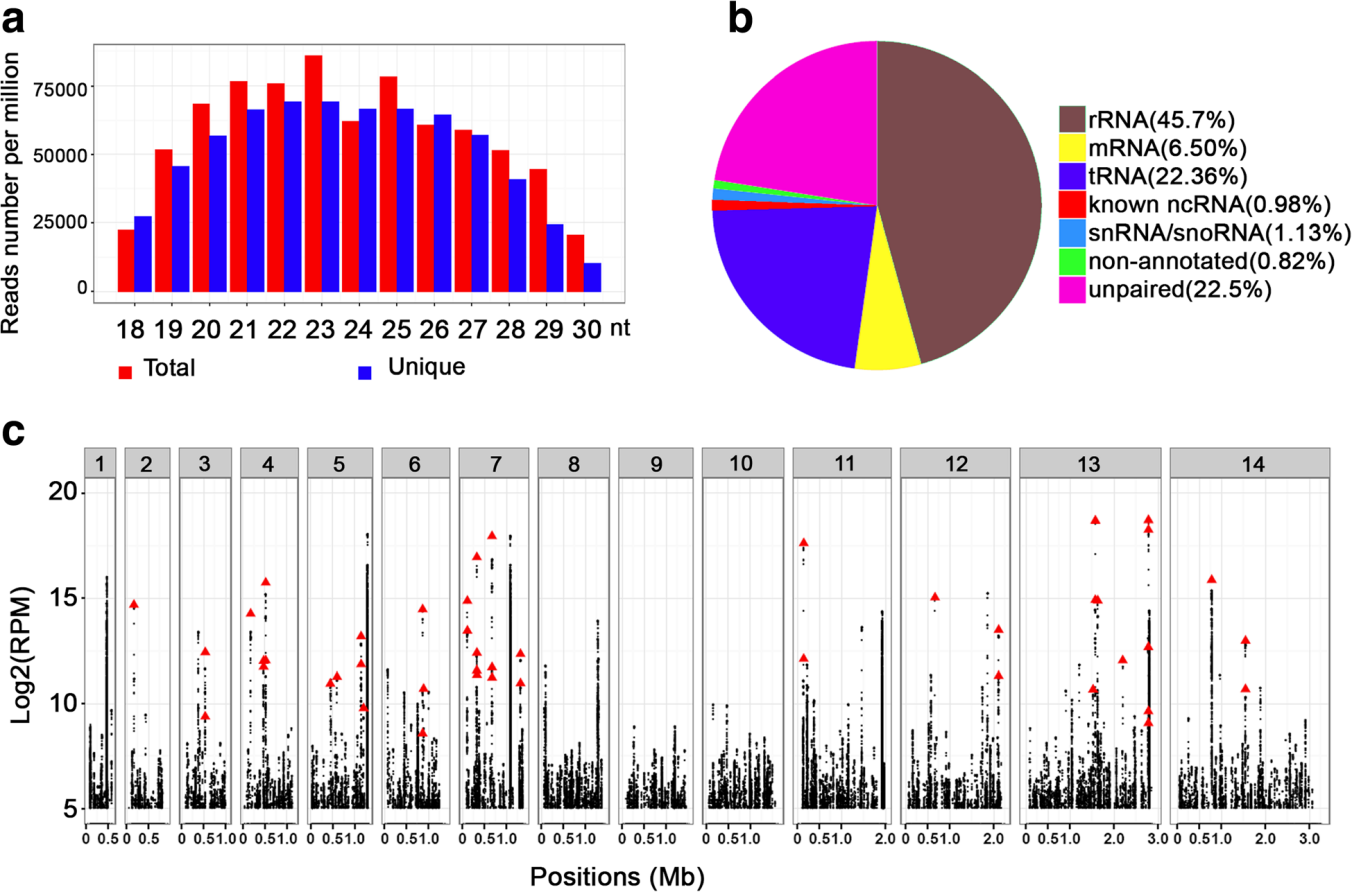
General features of 18–30 nt length small RNAs from *P. falciparum*. **a** Size distribution of the small RNAs derived from blood-stage parasites. **b** Classification and percentage of small RNA populations referring to the *P. falciparum* genome. **c** Schematic representation of the genome-wide density analysis for small RNAs on *P. falciparum* chromosomes. The read counts of total small RNAs were normalized to the relative number per one million reads (RPM) and plotted against the 14 parasite chromosomes. Red triangles show the locations and abundance of the *P. falciparum* tRNA-derived reads

The distribution of these small RNA reads appeared to be normal in length, with a peak at 23 nt for both total and unique RNAs, accounting for 12% of the total small RNAs and 11% of the unique RNAs, respectively (Fig. 1a). However, the slope of this peak is much lower than that for *Toxoplasma gondii*, an evolutionarily close apicomplexan parasite*.* Within the same range of 18–30 nt, small RNAs in *T. gondii* reached peaks at 21 and 26 nt in two different strains, in correspondence to 18.92% and 18.13% of unique small RNAs, respectively [31]. This implied that the features of *P. falciparum* small RNAs seemed to be different from the canonical 21–23 nt miRNAs. These *P. falciparum* small RNAs were derived from mRNAs, ribosomal RNAs (rRNAs), tRNAs, small nuclear RNA (snRNA)/small nucleolar RNAs (snoRNA), previously reported non-coding RNAs (ncRNAs), as well as some unannotated RNAs (Table 1, Fig. 1b). As expected, we found that the tRNA-derived small RNAs were abundantly presented in the library as the second predominant group, which represented 22.4% of the total reads (Fig. 1b). These small RNAs prevailed on all tRNA-originated chromosomes, while they were not found on chromosomes 1, 8, 9 and 10 due to the absence of tRNA genes on these chromosomes (Fig. 1c). Since only RNAs with a size of 18–30 nt were included in this study, shorter than those of mature tRNAs or tRNA-halves, and since quality control before sequencing limited their possibility of being RNA degradation residues during RNA processing, we predicted these tRNA-derived reads were *Plasmodium* tRFs.

### Identification of three types of tRFs from *P. falciparum*

**Fig. 2:**
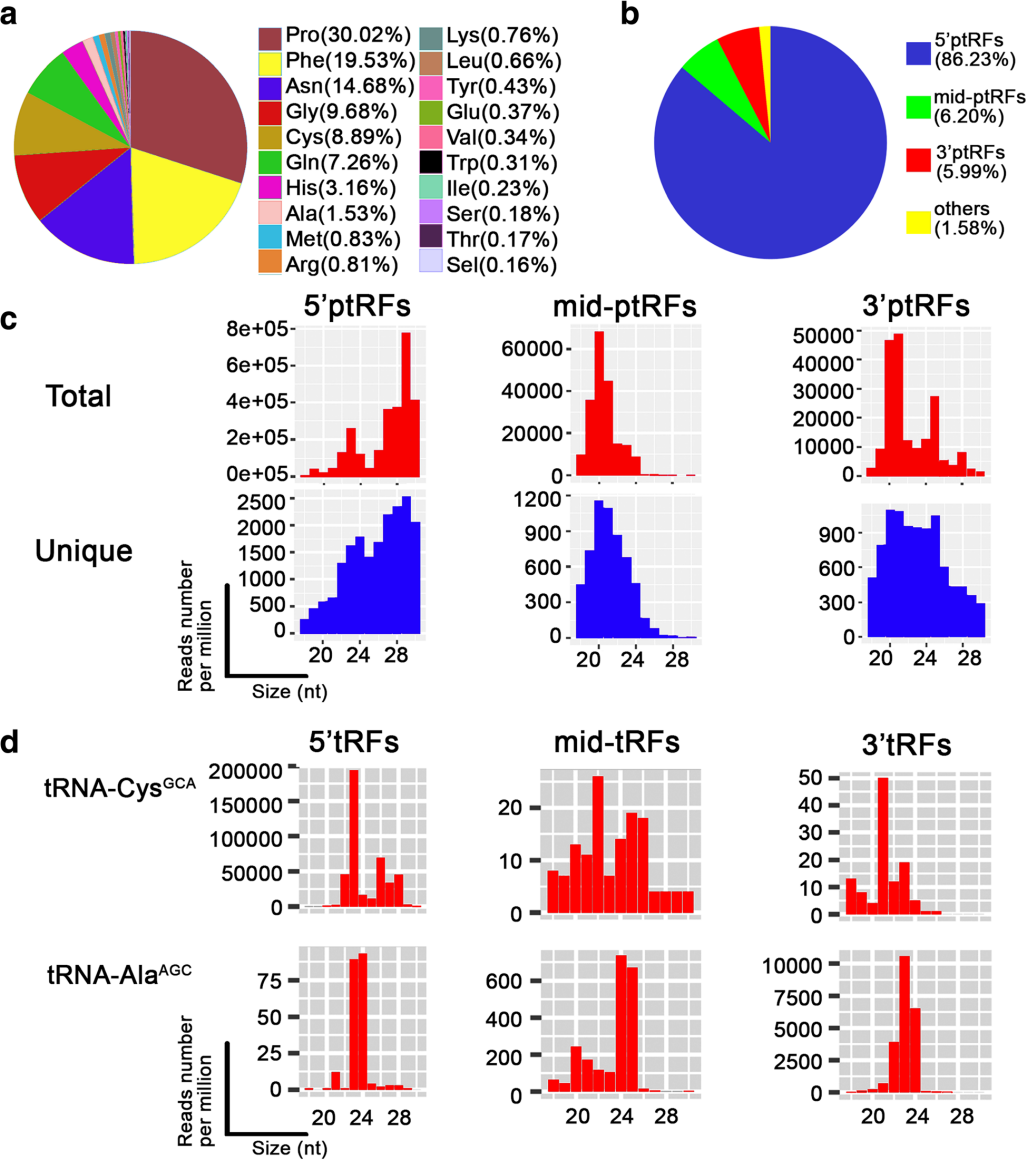
Distribution and abundance of 5'ptRFs, mid-ptRFs and 3'ptRFs. **a** ptRFs-originated tRNAs. **b** Frequency of ptRFs generated by tRNAs. **c** Size distribution of three ptRF types analyzed for their total and unique aspects. **d** Abundance analysis of three ptRF types derived from tRNA-Cys^GCA^ and tRNA-Ala^AGC^

We evaluated the amino acids coded by parental tRNAs of these ptRFs. The parental tRNAs of these ptRFs coded a total of 20 amino acids; however, 90% of the ptRFs were derived from tRNAs that coded eight amino acids: Pro, Phe, Asn, Gly, Cys, Gln, His and Ala (Fig. 2a). This bias further proved that these RNA fragments were not produced by random degeneration of mature tRNAs. We analyzed the RPM values of these fragments against their sizes and identified a distinct pattern in their size distribution, which showed two prominent peaks at ~23 and ~29 nt in correspondence to two groups (18–25 nt and 26–30 nt, respectively) (Additional file 2: Figure S1a).

Given that the biogenesis of tRFs from their parental tRNA molecules was reported to be evolutionarily conserved at the 5' end, 3' end and tRNA precursor, we mapped the ptRF reads to all 45 *Plasmodium* tRNA-coding genes and, as expected, we observed that a large portion of the reads fell into two groups that mapped to the 5' and 3' ends of their parent tRNAs. Unexpectedly, it was noticed that part of the ptRFs were mapped to the middle regions of the parent tRNAs, which were divergent in size and ends in contrast to the known mid-tRFs (or itRFs). Hence, we named these three ptRF types as “5'ptRFs”, “3'ptRFs” and “mid-ptRFs”. 5'ptRFs predominated (86.23%) in ptRFs, while mid-ptRFs and 3'ptRFs represented only 6.20% and 5.99% of the tRNA-derived fragments, respectively (Fig. 2b). By plotting the RPM values of three types of ptRFs against their sizes, we observed two peaks at ~23/24 and ~29 nt for 5'ptRFs, one peak at ~20 nt for mid-ptRFs, and two peaks at ~20/21 nt and ~25 nt for 3'ptRFs (Fig. 2c), which displayed great difference in the expression levels.

To further understand their expression divergence, we investigated the abundance of ptRFs derived from all 45 *P. falciparum* tRNAs. The RPM values of ptRFs in both total and unique categories were plotted against their sizes to create 270 plots (Additional files 3, 4 and 5: Figures S2, S3, S4), which exhibited great variance of the ptRFs from different tRNAs. Nine ptRFs with high RPM values over 10^5^ were observed, namely 5'ptRFs-Asp^GTC^, -Cys^GCA^, -Gly^GCC^, -His^GTG^, -Leu^CAA^, -Pro^AGG^, -Pro^CGG^, -Pro^TGG^ and 3'ptRFs-Asp^GTC^; whereas four ptRFs, 5'ptRFs-Sec^TCA^, mid-ptRFs-Arg^TCG^, -Leu^AAG^ and -Leu^TAG^ were found to have low RPM values less than 5 (Additional files 3, 4 and 5: Figures S2, S3, S4). For a given tRNA gene, the production of three types of ptRFs varied dramatically. For example, tRNA-Cys^GCA^ produced more 5'ptRFs than mid-ptRFs or 3'ptRFs with an abundance 3 × 10^3^-fold higher, while tRNA-Ala^AGC^ generated a lower level of 5'ptRFs than mid-ptRFs or 3'ptRFs (Fig. 2d). Consequently, no significant correlation was found within the abundance of these three types in the library. The Pearsonʼs correlation coefficient was -0.0176 (*P* = 0.6275) between 5'ptRFs and mid-ptRFs, 0.3192 between mid-ptRFs and 3'ptRFs (*P* = 0.3151), and 0.0218 between 3'ptRFs and 5'ptRFs (*P* = 0.8068) (Additional file 2: Figure S1b). We then selected the top 20 abundant ptRFs of each type to avoid any possibility of them being as RNA degradation products for further analysis (Additional file 1: Table S3). Interestingly, these three types of ptRFs were generally derived from various tRNAs, sharing only four common parental tRNAs (Additional file 2: Figure S1c). The generation of ptRFs did not seem to be associated with the expression levels of their parental tRNAs in *P. falciparum*.

### Experimental validation of ptRF candidates

**Fig. 3:**
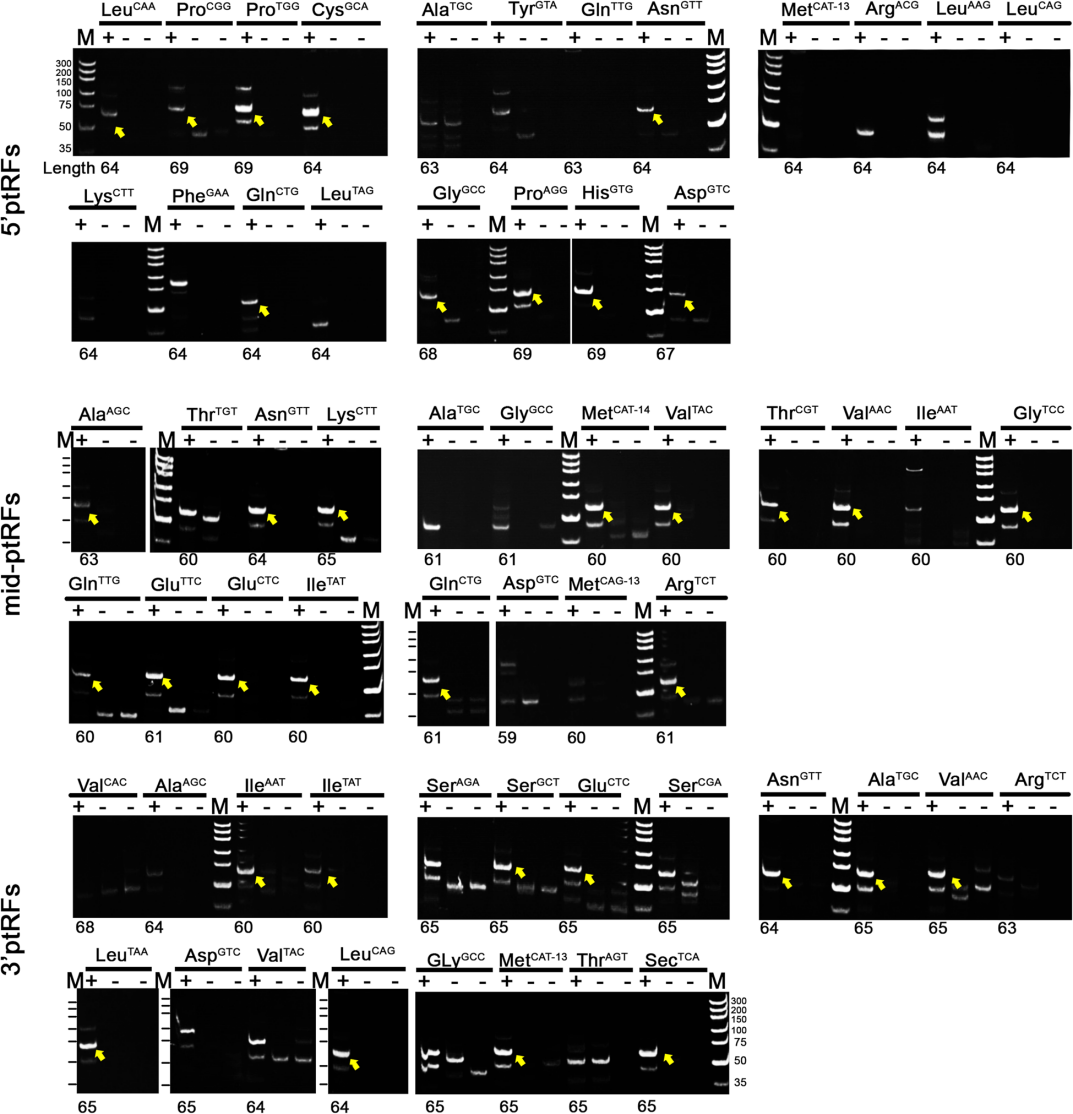
Validation of ptRFs by stem-loop RT-PCR. Each ptRF is shown in three adjacent lanes. From left to right represents stem-loop RT-PCR with *P. falciparum* RNA template (left, “+”), without RNA template (middle, “-”), or without reverse transcriptase (right, “-”), respectively. “M” indicates the DNA ladder. “Length” shows the expected size of PCR products, which is calculated by the size of each ptRF from the deep sequencing data plus 40 bp of nucleotides technically introduced by the design of specific stem-loop primers. The yellow arrow demonstrates the consistence of the PCR product size on the gel with the expected length

We experimentally evaluated the expression of the ptRFs in the transcriptome of *P. falciparum* to prove our findings. The top 20 abundant ptRFs of each type corresponding to 60 candidates were selected for stem-loop reverse transcription polymerase chain reaction (stem-loop RT-PCR). Thirty-five of these candidates (10 in 5'ptRFs, 14 in mid-ptRFs, and 11 in 3'ptRFs) were specifically amplified (Fig. 3), accounting for 58.3% of tested ptRFs. This confirmed the presence of tRFs in the blood stages of *P. falciparum*.

### New motifs found to process ptRFs at their cleavage sites

**Fig. 4:**
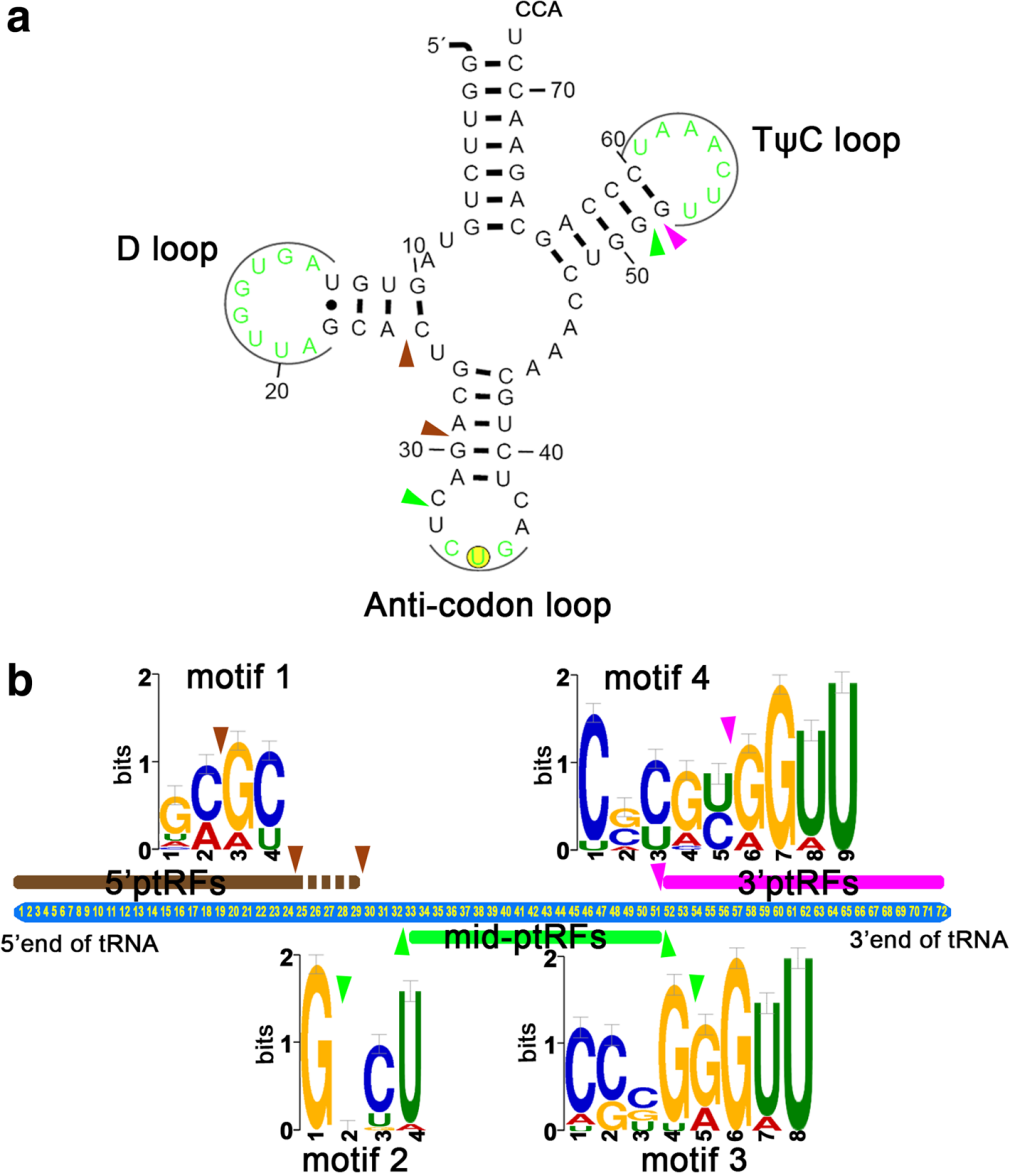
Cleavage sites and motif analysis of ptRFs. **a** Cleavage sites of ptRFs in mature tRNA, employing the secondary structure of tRNAs-Gln^CTG^ as an example. Brown arrowheads show the cleavage to generate 5'ptRFs, green arrowheads demonstrate the cleavage sites for mid-ptRFs, and pink arrowhead indicates the cleavage points for 3'ptRFs. **b** Schematic representation of the motif sequences and locations at the cleavage sites of the ptRFs. Arrowheads are the same as for panel **a**

To identify the cleavage sites, we matched each ptRF to their parental tRNA. As displayed in Fig. 4a, two cleavage sites were discovered to generate 5'ptRFs, with one occurring at the D-arm to produce 5'ptRFs of 23/24 nt, while one taking place at the anticodon stem to give rise to those with sizes around 29 nt. The mid-ptRFs originated from a combination of cleavage in the anticodon loop domain and TψC arm. However, the anticodon loop domain was cleaved at the 5' side instead of the middle section in this species, which was novel. Consequently, the cleavage in TψC arm generated the 3'ptRFs, and the variation of 3'CAA end in tRNAs resulted in two size groups (3'ptRFs-20/21 nt and 3'ptRFs-25 nt). We further explored motifs at the cleavage sites that might guide the generation of the ptRFs. Four motifs (motifs 1–4) were predicted to process ptRFs at each cleavage site (Fig. 4b). Although these motifs varied in sequence, they all had a signature of enriched G/C at the cleavage sites (Fig. 4b). Despite the fact that the 5'ptRFs showed two sub-classes, one common motif seemed to be responsible for the cleavage. Through comparison analysis, we sought to determine whether these motifs matched with the known motifs in the database including 24 species and 244 predicted RNA motifs [32] and found no motifs showing high homology with those reported here, indicating these four motifs were first identified in *P. falciparum*.

## Discussion

In spite of the progress made in post-genomic research on malaria parasites, our knowledge on the function of small non-coding RNAs in these organisms remains insufficient. The limitation in cloning techniques to identify small RNAs in *P. falciparum* has restricted genome-wide small RNA characterization. Yet, high-throughput sequencing provides advanced depth and massive volumes, thereby enables the exploration of distinct categories of RNAs compared with traditional methods [19, 20]. It is widely accepted that miRNAs are not the only regulators of gene expression; many other types of small RNAs have been identified to contribute to this process. tRFs is one such type, associated with many functions similar to miRNAs at the post-transcriptional level. Thus, it is possible that *Plasmodium* parasites complete gene expression regulation through molecules that can substitute for miRNAs, e.g. tRFs. Studies have shown that tRF expression is evolutionarily conserved from prokaryotes to mammalian cells. In the present study, we took advantage of high-throughput sequencing to obtain a coverage over 100-fold for 18–30 nt length small RNAs, which provided sufficient depth for systematic analysis. Consistent with tRFs derived from plant or mammalian cell lines [13, 33], we found three types of tRFs in *P. falciparum*: 5'ptRFs, 3'ptRFs and mid-ptRFs. Of these, 5'ptRFs is the most abundant class, while mid-ptRFs are generated from the 5' end of the anticodon loop of their parental tRNAs, which has not been previously reported [12, 34, 35].

The mechanism of tRF biogenesis has not been understood clearly because cleavage sites of known tRNA-specific nucleases remain ambiguous [36]. Information on the consensus feature(s) of cleavage sites on tRNAs needs to be fully addressed [37]. Therefore, we analyzed the motifs for the *P. falciparum* tRNAs to produce tRFs, and discovered four new motifs at the ends of the ptRFs that may aid the explanation of the versatile production of ptRFs. Intriguingly, motif 3 at the 3' end of the mid-ptRFs and motif 4 at the 5' end of the 3'ptRFs were found from different tRNA molecules in our analysis, while they shared analogous sequences. We noticed that these motifs are also situated at the same sites in those tRNAs not producing ptRFs. It is possible that the generation of ptRFs might involve multiple factors in addition to motif recognition. Moreover, the D-loop adjacent to motif 1 contains the “GG” conserved dinucleotide, which is required by RNase P and 39-tRNase processing and is highly conserved in *Drosophila* and humans [38]. Collectively, these findings provide a clue that there might be unidentified *Plasmodium*-derived endonucleases playing roles in the biogenesis of ptRFs.

The functional proteins closely associated with tRFs are reported to be DICER, AGO1, 2, 4 and 5 in plant cells, and AGO1, 3 and 4 in animal cells [13, 14, 33]. Yet, genome sequencing of *P. falciparum* has not revealed the existence of DICER and AGO homologs. Thus, the mechanism of function and related proteins for ptRFs remains to be elucidated. Evidence from one previous study indicates that human miRNAs complexed with human AGO2 are translocated into the parasite cytoplasm to function as negative regulators of the infection [39]. Host-derived miRNA molecules have been shown to interfere with the synthesis of *Plasmodium* proteins [30]. Whether ptRFs function by binding to host AGO2 or other small RNA biogenesis-related apparatus should be further explored to strengthen our understanding of the interaction between malaria parasites and their human host.

In *P. berghei* and its closely related species *T. gondii*, half-tRNAs with a size of nearly 35 nt have been detected experimentally [40]. Although stress-induced tRNA-halves are not included in this study, they need to be explored in *P. falciparum*. Therefore, more work is required to investigate ptRFs and other small RNAs as well as their function both *in vitro* and *in vivo.*

## Conclusions

To our knowledge, this is the first comprehensive analysis of tRFs in *P. falciparum*. Three types of tRFs were identified, and four new motifs guiding their cleavage were revealed. The generation and function of these ptRFs need further exploration. Our work provides evidence of tRFs in *P. falciparum*, which could promote research on the functions of tRFs in the pathogenesis of *Plasmodium* infections and potentially lead to new therapeutic approaches to malarial disease.

## Additional files

Additional file 1: **Table S1.** Sequences of specific stem-loop reverse transcription primers. **Table S2.** Sequences of PCR primers for stem-loop RT-PCR. **Table S3.** RPM values and sizes of the top 20 abundant ptRFs for each type. (DOCX 41 kb)

Additional file 2: **Figure S1.** Abundance and correlation analysis of ptRFs. **a** Size distribution of ptRFs in total and unique categories. **b** Correlation of parental tRNAs of three types of ptRFs. Each dot represents a tRNA; R value indicates the Pearsonʼs correlation coefficient. **c** Common parental tRNAs for the top 20 ptRFs of each type showing in Venn diagrams. (TIF 535 kb)

Additional file 3: **Figure S2.** Size distributions of the 5'ptRFs in total and unique aspects. Solid boxes represent those with RPMs above 10^5^ and dotted boxes represent those with RPMs less than 5. (TIF 2602 kb)

Additional file 4: **Figure S3.** Size distributions of the mid-ptRFs in total and unique aspects. Dotted boxes correspond to those with RPMs less than 5. (TIF 2578 kb)

Additional file 5: **Figure S4.** Size distribution of the 3'ptRFs in total and unique aspects. The solid box symbolizes those with RPMs above 10^5^. (TIF 2626 kb)

## Abbreviations

AGO: Argonaute protein
cDNA: Complementary DNA
DNA: Deoxyribonucleic acid
endo-siRNA: Endogenous small interference RNA
gDNA: Genomic DNA
miRNA: MicroRNA
ncRNA: Non-coding RNA
Pf3D7: *P. falciparum* laboratory strain 3D7
piRNA: PiWi-interacting RNA
PiWi: P-element induced wimpy testis
ptRFs: *Plasmodium* tRFs
RBCs: Red blood cells
RNA: Ribonucleic acid
RPM: Relative number per one million reads
RPMI-1640: Roswell Park Memorial Institute-1640
rRNA: Ribosomal RNA
RT-PCR: Quantitative reverse-transcribed polymerase chain reaction
snoRNA: Small nucleolar RNA
snRNA: Small nuclear RNA
tRFs: Transfer RNA-derived fragments
tRNA: Transfer RNA
