# Supplementary material for: Genome-wide identification and characterization of transfer RNA-derived small RNAs in Plasmodium falciparum
Source: Parasit Vectors. 2019 Jan 15;12:36. doi: 10.1186/s13071-019-3301-6 (PMC6332904; doi:10.1186/s13071-019-3301-6)
Supplement: Supplementary file 1 — Table S1. Sequences of specific stem-loop reverse transcription primers. Table S2. Sequences of PCR primers for stem-loop RT-PCR. Table S3. RPM values and sizes of the top 20 abundant ptRFs for each type. (DOCX 41 kb) [file 13071_2019_3301_MOESM1_ESM.docx]

**Genome-wide identification and characterization of transfer RNA-derived small RNAs in *Plasmodium falciparum***

Zhensheng Wang^1^, Chunyan Wei^1^, Xiao Hao^1^, Weiwei Deng^1^, Lianhui Zhang^1^, Zenglei Wang^2^*, Heng Wang^1^*

1. Department of Microbiology and Parasitology, Institute of Basic Medical Sciences Chinese Academy of Medical Sciences, School of Basic Medicine Peking Union Medical College, 5# Dong Dan San Tiao, Beijing, 100005, P. R. China

2. NHC Key Laboratory of Systems Biology of Pathogens, Institute of Pathogen Biology, Chinese Academy of Medical Sciences & Peking Union Medical College, Beijing, P. R. China

*Correspondence: zengleiwang@pumc.edu.cn, [wangh@ibms.cams.cn](mailto:wangh@ibms.cams.cn)

E-mails:

Zhensheng Wang: [wangzs@](mailto:negen@126.com)ibms.pumc.edu.cn

Chunyan Wei: [weichunyan2004@126.com](mailto:weichunyan2004@126.com)

Xiao Hao: haoxiao7777@126.com

Weiwei Deng: [vivian_dengww2007@163.com](mailto:vivian_dengww2007@163.com)

Lianhui Zhang: [zhanglianhuipumc@126.com](mailto:zhanglianhuipumc@126.com)

Zenglei Wang: [zengleiwang@pumc.edu.cn](mailto:zengleiwang@pumc.edu.cn)

Heng Wang: [wangh@ibms.cams.cn](mailto:wangh@ibms.cams.cn)

**Additional file 1: Table S1.** The sequences of specific stem-loop reverse transcription primers

| Name | Sequences | |
| --- | --- | --- |
| Pro^TGG^5trfRT | | *GTCGTATCCAGTGCAGGGTCCGAGGTTCGCACTGGATACGAC*GCGAGAATC |
| Pro^AGG^5trfRT | | *GTCGTATCCAGTGCAGGGTCCGAGGTTCGCACTGGATACGAC*GAGAGAATC |
| Pro^CGG^5trfRT | | *GTCGTATCCAGTGCAGGGTCCGAGGTTCGCACTGGATACGAC*GCAAGAATC |
| Cys^GCA^5trfRT | | *GTCGTATCCAGTGCAGGGTCCGAGGTTCGCACTGGATACGAC*GCTCTAC |
| Gly^GCC^5trfRT | | *GTCGTATCCAGTGCAGGGTCCGAGGTTCGCACTGGATACGAC*GAAGTATTC |
| Leu^CAA^5trfRT | | *GTCGTATCCAGTGCAGGGTCCGAGGTTCGCACTGGATACGAC*TTTAGACC |
| His^GTG^5trfRT | | *GTCGTATCCAGTGCAGGGTCCGAGGTTCGCACTGGATACGAC*TGGAGTCC |
| Asp^GTC^5trfRT | | *GTCGTATCCAGTGCAGGGTCCGAGGTTCGCACTGGATACGAC*GAAATAC |
| Ala^TGC^5trfRT | | *GTCGTATCCAGTGCAGGGTCCGAGGTTCGCACTGGATACGAC*TGATACCAC |
| Tyr^GTA^5trfRT | | *GTCGTATCCAGTGCAGGGTCCGAGGTTCGCACTGGATACGAC*GCTCTAC |
| Gln^TTG^5trfRT | | *GTCGTATCCAGTGCAGGGTCCGAGGTTCGCACTGGATACGAC*GTGCTAAC |
| Asn^GTT^5trfRT | | *GTCGTATCCAGTGCAGGGTCCGAGGTTCGCACTGGATACGAC*CTCTAAC |
| Met^CAT-13^5trfRT | | *GTCGTATCCAGTGCAGGGTCCGAGGTTCGCACTGGATACGAC*ACTCTTC |
| Arg^ACG^5trfRT | | *GTCGTATCCAGTGCAGGGTCCGAGGTTCGCACTGGATACGAC*TCTATCC |
| Leu^AAG^5trfRT | | *GTCGTATCCAGTGCAGGGTCCGAGGTTCGCACTGGATACGAC*CTTAGAC |
| Leu^CAG^5trfRT | | *GTCGTATCCAGTGCAGGGTCCGAGGTTCGCACTGGATACGAC*CTTAGAC |
| Lys^CTT^5trfRT | | *GTCGTATCCAGTGCAGGGTCCGAGGTTCGCACTGGATACGAC*CTCTACC |
| Phe^GAA^5trfRT | | *GTCGTATCCAGTGCAGGGTCCGAGGTTCGCACTGGATACGAC*CTCTCCC |
| Gln^CTG^5trfRT | | *GTCGTATCCAGTGCAGGGTCCGAGGTTCGCACTGGATACGAC*GTGCTAAC |
| Leu^TAG^5trfRT | | *GTCGTATCCAGTGCAGGGTCCGAGGTTCGCACTGGATACGAC*CTTAGAC |
| Asp^GTC^MtrfRT | | *GTCGTATCCAGTGCAGGGTCCGAGGTTCGCACTGGATACGAC*CGGGTCTTCC |
| Arg^TCT^MtrfRT | | *GTCGTATCCAGTGCAGGGTCCGAGGTTCGCACTGGATACGAC*CGCAGC |
| Gln^CTG^MtrfRT | | *GTCGTATCCAGTGCAGGGTCCGAGGTTCGCACTGGATACGAC*CCAGGTTTG |
| Ala^AGC^MtrfRT | | *GTCGTATCCAGTGCAGGGTCCGAGGTTCGCACTGGATACGAC*CGTACCTC |
| Met^CAT-13^MtrfRT | | *GTCGTATCCAGTGCAGGGTCCGAGGTTCGCACTGGATACGAC*ACGGTCCTG |
| Ala^TGC^MtrfRT | | *GTCGTATCCAGTGCAGGGTCCGAGGTTCGCACTGGATACGAC*CCGGACC |
| Gly^GCC^MtrfRT | | *GTCGTATCCAGTGCAGGGTCCGAGGTTCGCACTGGATACGAC*CGGGTCAC |
| Met^CAT-14^MtrfRT | | *GTCGTATCCAGTGCAGGGTCCGAGGTTCGCACTGGATACGAC*CACGACC |
| Val^TAC^MtrfRT | | *GTCGTATCCAGTGCAGGGTCCGAGGTTCGCACTGGATACGAC*CGGGATC |
| Thr^CGT^MtrfRT | | *GTCGTATCCAGTGCAGGGTCCGAGGTTCGCACTGGATACGAC*CACGATC |
| Val^AAC^MtrfRT | | *GTCGTATCCAGTGCAGGGTCCGAGGTTCGCACTGGATACGAC*GGGATCTC |
| Ile^AAT^MtrfRT | | *GTCGTATCCAGTGCAGGGTCCGAGGTTCGCACTGGATACGAC*ACCTTAC |
| Gly^TCC^MtrfRT | | *GTCGTATCCAGTGCAGGGTCCGAGGTTCGCACTGGATACGAC*CGGGTCAC |
| Gln^TTG^MtrfRT | | *GTCGTATCCAGTGCAGGGTCCGAGGTTCGCACTGGATACGAC*CGGGTTGG |
| Glu^TTC^MtrfRT | | *GTCGTATCCAGTGCAGGGTCCGAGGTTCGCACTGGATACGAC*CGGGTCG |
| Glu^CTC^MtrfRT | | *GTCGTATCCAGTGCAGGGTCCGAGGTTCGCACTGGATACGAC*CGGGCCT |
| Ile^TAT^MtrfRT | | *GTCGTATCCAGTGCAGGGTCCGAGGTTCGCACTGGATACGAC*CACGACC |
| Thr^TGT^MtrfRT | | *GTCGTATCCAGTGCAGGGTCCGAGGTTCGCACTGGATACGAC*CCCGACC |
| Asn^GTT^MtrfRT | | *GTCGTATCCAGTGCAGGGTCCGAGGTTCGCACTGGATACGAC*ACCAACG |
| Lys^CTT^MtrfRT | | *GTCGTATCCAGTGCAGGGTCCGAGGTTCGCACTGGATACGAC*CCCTGACC |
| Asp^GTC^3trfRT | | *GTCGTATCCAGTGCAGGGTCCGAGGTTCGCACTGGATACGAC*TGGCTCC |
| Leu^TAA^3trfRT | | *GTCGTATCCAGTGCAGGGTCCGAGGTTCGCACTGGATACGAC*TGGTGC |
| Val^TAC^3trfRT | | *GTCGTATCCAGTGCAGGGTCCGAGGTTCGCACTGGATACGAC*TGGTACG |
| Leu^CAG^3trfRT | | *GTCGTATCCAGTGCAGGGTCCGAGGTTCGCACTGGATACGAC*CACGCCT |
| Asn^GTT^3trfRT | | *GTCGTATCCAGTGCAGGGTCCGAGGTTCGCACTGGATACGAC*TGGGATC |
| Ala^TGC^3trfRT | | *GTCGTATCCAGTGCAGGGTCCGAGGTTCGCACTGGATACGAC*TGGTGGAC |
| Val^AAC^3trfRT | | *GTCGTATCCAGTGCAGGGTCCGAGGTTCGCACTGGATACGAC*TGGTACG |
| Arg^TCT^3trfRT | | *GTCGTATCCAGTGCAGGGTCCGAGGTTCGCACTGGATACGAC*GGATTCG |
| Val^CAC^3trfRT | | *GTCGTATCCAGTGCAGGGTCCGAGGTTCGCACTGGATACGAC*TGGTTAAT |
| Ala^AGC^3trfRT | | *GTCGTATCCAGTGCAGGGTCCGAGGTTCGCACTGGATACGAC*TGGACGAC |
| Ile^AAT^3trfRT | | *GTCGTATCCAGTGCAGGGTCCGAGGTTCGCACTGGATACGAC*TTTGGTCC |
| Ile^TAT^3trfRT | | *GTCGTATCCAGTGCAGGGTCCGAGGTTCGCACTGGATACGAC*TGGTGATC |
| Ser^AGA^3trfRT | | *GTCGTATCCAGTGCAGGGTCCGAGGTTCGCACTGGATACGAC*TGGCGAC |
| Ser^GCT^3trfRT | | *GTCGTATCCAGTGCAGGGTCCGAGGTTCGCACTGGATACGAC*CGACAACG |
| Glu^CTC^3trfRT | | *GTCGTATCCAGTGCAGGGTCCGAGGTTCGCACTGGATACGAC*TGGTTCC |
| Ser^CGA^3trfRT | | *GTCGTATCCAGTGCAGGGTCCGAGGTTCGCACTGGATACGAC*TGGCGAC |
| Gly^GCC^3trfRT | | *GTCGTATCCAGTGCAGGGTCCGAGGTTCGCACTGGATACGAC*TGGTGC |
| Met^CAT-13^3trfRT | | *GTCGTATCCAGTGCAGGGTCCGAGGTTCGCACTGGATACGAC*TGGTAGC |
| Thr^AGT^3trfRT | | *GTCGTATCCAGTGCAGGGTCCGAGGTTCGCACTGGATACGAC*TGGAGCC |
| Sec^TCA^3trfRT | | *GTCGTATCCAGTGCAGGGTCCGAGGTTCGCACTGGATACGAC*CACGACC |

The italic letters represent the common stem-loop sequence which ensure the specificity of the reverse transcription.

**Additional file 1: Table S2.** The sequences of PCR primers in the stem-loop RT-PCR

| Name | Sequences | |
| --- | --- | --- |
| Pro^TGG^5trfF | | GACTGGCTACTTAGTCTAGTG |
| Pro^AGG^5trfF | | GACTGGCTACTTAGTCTAGTG |
| Pro^CGG^5trfF | | GACTGGCTACTTGATCTAGTG |
| Cys^GCA^5trfF | | TAATGGGCGTGTAGCTCAGC |
| Gly^GCC^5trfF | | GACTGCATCTGTGGTCTAGTG |
| Leu^CAA^5trfF | | GACTGCACGGATGGCTGAG |
| His^GTG^5trfF | | GACTGTCCAAATCGTCTAG |
| Asp^GTC^5trfF | | GACTTCCGAGATAGTATAG |
| Ala^TGC^5trfF | | GACTGGGCAGGTGGTGTAG |
| Tyr^GTA^5trfF | | GACTCCGATGATAGCTCAG |
| Gln^TTG^5trfF | | GACTGGTTTCGTAGTGTAG |
| Asn^GTT^5trfF | | GACTGGTTCCGTAGCTCAG |
| Met^CAT-13^5trfF | | GACTAGCAGCGTAGCTCAG |
| Arg^ACG^5trfF | | GACTGGGCCGGTAGTTCA |
| Leu^AAG^5trfF | | GACTGACAGAATGGCCGA |
| Leu^CAG^5trfF | | GACTGACAGAATGGCCGAG |
| Lys^CTT^5trfF | | GACTGCTGCCTTAGCTCAG |
| Phe^GAA^5trfF | | GACTGCCGTGATAGCTCAGT |
| Gln^CTG^5trfF | | GACTGGTTCTGTAGTGTAG |
| Leu^TAG^5trfF | | GACTGTCAGGATGGCCGAG |
| Asp^GTC^MtrfF | | GACTTACGAGTCGTAGAGTG |
| Arg^TCT^MtrfF | | GACTACTTCTAATCCAAAG |
| Gln^CTG^MtrfF | | GACTACTCTGACTCTGC |
| Ala^AGC^MtrfF | | GACTGCTTAGCATGCGAG |
| Met^CAT-13^MtrfF | | GACTCTCATAACCCCCAG |
| Ala^TGC^MtrfF | | GACTATTTGCATTCAAG |
| Gly^GCC^MtrfF | | GACTGTTGCCATCGAAG |
| Met^CAT-14^MtrfF | | GACTCTCATAATCCCGA |
| Val^TAC^MtrfF | | GACTCTTACACGCAGG |
| Thr^CGT^MtrfF | | GACTTTCGTAATCAAAAG |
| Val^AAC^MtrfF | | GACTGCCTAACACGCAG |
| Ile^AAT^MtrfF | | GACTACGGCTAATAACC |
| Gly^TCC^MtrfF | | GACTCCTTCCAAGCAG |
| Gln^TTG^MtrfF | | GACTACTTTGAATCCTC |
| Glu^TTC^MtrfF | | GACTCTTTCACCCGAAC |
| Glu^CTC^MtrfF | | GACTGCTCTCACCCGAAAG |
| Ile^TAT^MtrfF | | GACTCTTATGTACCGAAG |
| Thr^TGT^MtrfF | | GACTCTTGTAAACCAAAG |
| Asn^GTT^MtrfF | | GACTGCTGTTAACCGCAAG |
| Lys^CTT^MtrfF | | GACTCAGACTCTTAATCTG |
| Asp^GTC^3trfF | | GACTGGTTCAATTCCCGGTC |
| Leu^TAA^3trfF | | GACTGGTTCGAACCCCACC |
| Val^TAC^3trfF | | GACTGTTCGATCCTCGGTG |
| Leu^CAG^3trfF | | GACTAGATTGTAGTCTGTAT |
| Asn^GTT^3trfF | | GACTCGCAAGGTCGTTGG |
| Ala^TGC^3trfF | | GACTGGTTCAATTCCCCGTC |
| Val^AAC^3trfF | | GACTGTTCGATCCTCGGTGC |
| Arg^TCT^3trfF | | GACTAAGGCTGCGGGTTC |
| Val^CAC^3trfF | | GACTGTTCGATCCTCGGTG |
| Ala^AGC^3trfF | | GACTGATCGATACCCCGGTC |
| Ile^AAT^3trfF | | GACTGGTTCGAGACCGCCTG |
| Ile^TAT^3trfF | | GACTGGTTCGAAACCCACA |
| Ser^AGA^3trfF | | GACTGGTTCGAATCCTGC |
| Ser^GCT^3trfF | | GACTGGTTCGATTCCTGC |
| Glu^CTC^3trfF | | GACTGGTTCAATTCCC |
| Ser^CGA^3trfF | | GACTGGTTCAAATCCTGTAG |
| Gly^GCC^3trfF | | GACTGGTTCGATTCCCGGCA |
| Met^CAT-13^3trfF | | GACTGGATCGAAACCACG |
| Thr^AGT^3trfF | | GACTGGTTCGACTCCGGTTG |
| Sec^TCA^3trfF | | GACTCTCATAATCCCGAG |
| General R* | | TCCAGTGCAGGGTCCGAGG |

* “General” R is a common reverse primer designed from the sequence of stem-loop structure in reverse transcription primers in Table 1.

**Additional file 1: Table S3.** RPM data and sizes of the top 20 ptRFs for each type

| The type of ptRFs | The parent tRNA | Gene ID | RPM | Size (nt) |
| --- | --- | --- | --- | --- |
| 5’ptRFs | Pro^TGG^ | PF3D7_1339200 | 511804 | 29 |
| 5’ptRFs | Pro^AGG^ | PF3D7_1418400 | 508152 | 29 |
| 5’ptRFs | Pro^CGG^ | PF3D7_1216800 | 426831 | 29 |
| 5’ptRFs | Cys^GCA^ | PF3D7_1370100 | 421989 | 24 |
| 5’ptRFs | Gly^GCC^ | PF3D7_1370200 | 309701 | 28 |
| 5’ptRFs | Leu^CAA^ | PF3D7_1103300 | 193466 | 24 |
| 5’ptRFs | His^GTG^ | PF3D7_706900 | 142473 | 29 |
| 5’ptRFs | Asp^GTC^ | PF3D7_714700 | 117596 | 27 |
| 5’ptRFs | Ala^TGC^ | PF3D7_411500 | 28458 | 23 |
| 5’ptRFs | Tyr^GTA^ | PF3D7_702800 | 9313 | 24 |
| 5’ptRFs | Gln^TTG^ | PF3D7_1252000 | 8876 | 23 |
| 5’ptRFs | Asn^GTT^ | PF3D7_403000 | 8543 | 24 |
| 5’ptRFs | Met^CAT-13^ | PF3D7_1339100 | 6393 | 24 |
| 5’ptRFs | Arg^ACG^ | PF3D7_1369800 | 5823 | 24 |
| 5’ptRFs | Leu^AAG^ | PF3D7_714800 | 4221 | 24 |
| 5’ptRFs | Leu^CAG^ | PF3D7_620900 | 4119 | 24 |
| 5’ptRFs | Lys^CTT^ | PF3D7_707000 | 3201 | 24 |
| 5’ptRFs | Phe^GAA^ | PF3D7_514400 | 2127 | 24 |
| 5’ptRFs | Gln^CTG^ | PF3D7_203500 | 2100 | 24 |
| 5’ptRFs | Leu^TAG^ | PF3D7_510600 | 1872 | 24 |
| Mid-ptRFs | Asp^GTC^ | PF3D7_714700 | 39884 | 19 |
| Mid-ptRFs | Arg^TCT^ | PF3D7_1341000 | 28481 | 21 |
| Mid-ptRFs | Gln^CTG^ | PF3D7_203500 | 24177 | 21 |
| Mid-ptRFs | Ala^AGC^ | PF3D7_702700 | 22185 | 23 |
| Mid-ptRFs | Met^CAT-13^ | PF3D7_1339100 | 21561 | 20 |
| Mid-ptRFs | Ala^TGC^ | PF3D7_411500 | 17036 | 21 |
| Mid-ptRFs | Gly^GCC^ | PF3D7_1370200 | 8580 | 21 |
| Mid-ptRFs | Met^CAT-14^ | PF3D7_1438300 | 6386 | 20 |
| Mid-ptRFs | Val^TAC^ | PF3D7_730600 | 3458 | 20 |
| Mid-ptRFs | Thr^CGT^ | PF3D7_1355400 | 3269 | 20 |
| Mid-ptRFs | Val^AAC^ | PF3D7_1251900 | 2738 | 20 |
| Mid-ptRFs | Ile^AAT^ | PF3D7_312700 | 2593 | 20 |
| Mid-ptRFs | Gly^TCC^ | PF3D7_1103200 | 2021 | 20 |
| Mid-ptRFs | Gln^TTG^ | PF3D7_1252000 | 1897 | 20 |
| Mid-ptRFs | Glu^TTC^ | PF3D7_527700 | 1846 | 21 |
| Mid-ptRFs | Glu^CTC^ | PF3D7_411600 | 1521 | 20 |
| Mid-ptRFs | Ile^TAT^ | PF3D7_410200 | 1252 | 20 |
| Mid-ptRFs | Thr^TGT^ | PF3D7_730700 | 1213 | 20 |
| Mid-ptRFs | Asn^GTT^ | PF3D7_403000 | 1129 | 24 |
| Mid-ptRFs | Lys^CTT^ | PF3D7_707000 | 1024 | 25 |
| 3’ptRFs | Asp^GTC^ | PF3D7_714700 | 109028 | 25 |
| 3’ptRFs | Leu^CAG^ | PF3D7_620900 | 21119 | 25 |
| 3’ptRFs | Asn^GTT^ | PF3D7_403000 | 10023 | 24 |
| 3’ptRFs | Leu^TAA^ | PF3D7_527800 | 3253 | 24 |
| 3’ptRFs | Ala^TGC^ | PF3D7_411500 | 3232 | 24 |
| 3’ptRFs | Val^TAC^ | PF3D7_730600 | 3099 | 25 |
| 3’ptRFs | Val^AAC^ | PF3D7_1251900 | 3090 | 25 |
| 3’ptRFs | Arg^TCT^ | PF3D7_1341000 | 2812 | 23 |
| 3’ptRFs | Val^CAC^ | PF3D7_312600 | 2757 | 28 |
| 3’ptRFs | Ala^AGC^ | PF3D7_702700 | 2170 | 24 |
| 3’ptRFs | Ile^AAT^ | PF3D7_312700 | 2076 | 20 |
| 3’ptRFs | Ile^TAT^ | PF3D7_410200 | 2035 | 20 |
| 3’ptRFs | Ser^AGA^ | PF3D7_410100 | 1995 | 20 |
| 3’ptRFs | Ser^GCT^ | PF3D7_714900 | 1858 | 25 |
| 3’ptRFs | Glu^CTC^ | PF3D7_411600 | 1835 | 25 |
| 3’ptRFs | Ser^CGA^ | PF3D7_1337600 | 1594 | 25 |
| 3’ptRFs | Gly^GCC^ | PF3D7_1370200 | 1550 | 25 |
| 3’ptRFs | Met^CAT-13^ | PF3D7_1339100 | 1522 | 25 |
| 3’ptRFs | Thr^AGT^ | PF3D7_706800 | 1468 | 25 |
| 3’ptRFs | Sec^TCA^ | PF3D7_1438200 | 1288 | 20 |

“Gene ID” is referred to PlasmoDB (http://plasmodb.org/plasmo/); RPM represents a **r**elative number **p**er one **m**illion reads; size stands for the length in number of nucleotide.
