# Supplementary figures and images for: Genome-wide identification and characterization of transfer RNA-derived small RNAs in Plasmodium falciparum
Source: Parasit Vectors. 2019 Jan 15;12:36. doi: 10.1186/s13071-019-3301-6 (PMC6332904; doi:10.1186/s13071-019-3301-6)

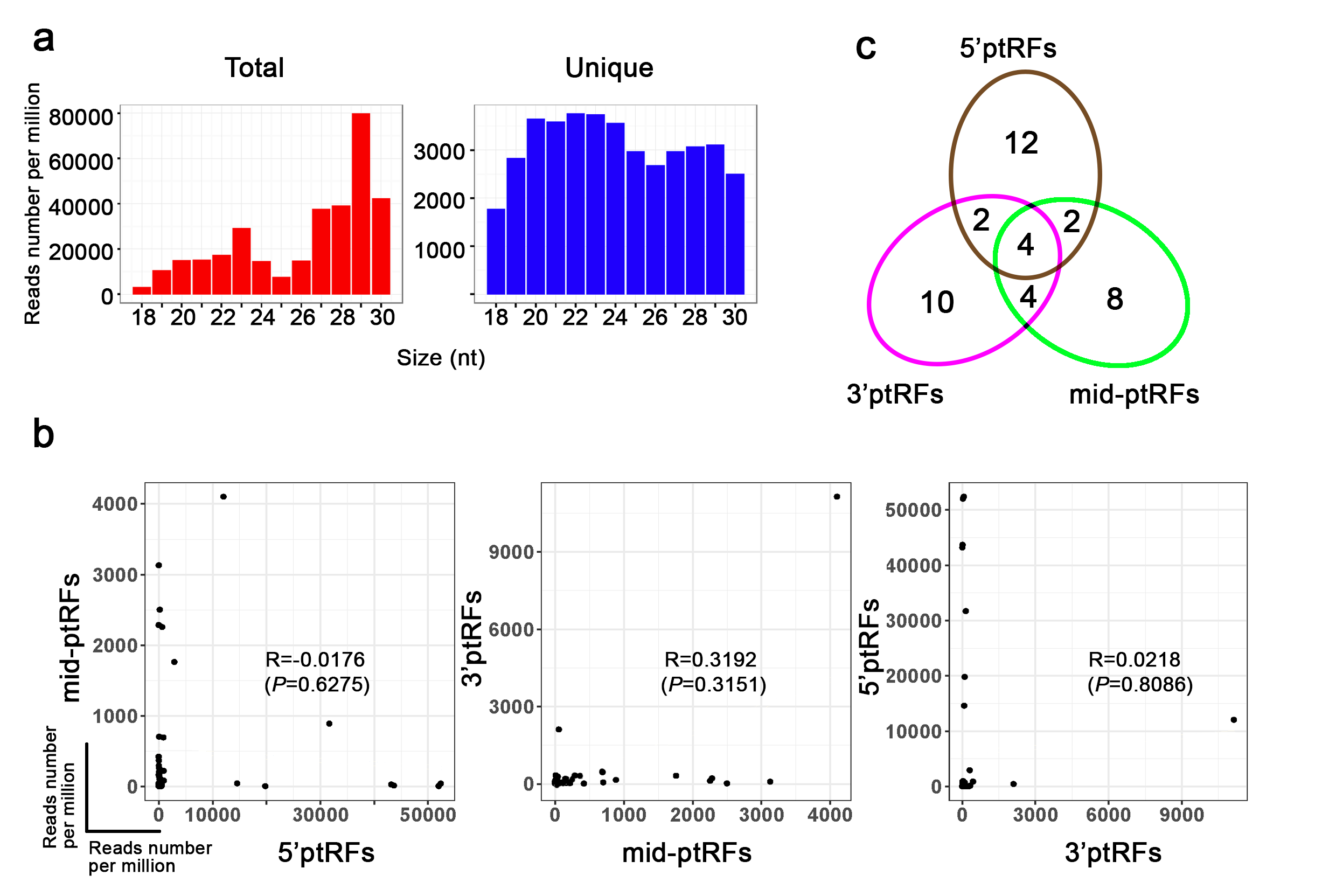

Supplement: Supplementary file 2 — Figure S1. Abundance and correlation analysis of ptRFs. a Size distribution of ptRFs in total and unique categories. b Correlation of parental tRNAs of three types of ptRFs. Each dot represents a tRNA; R value indicates the Pearsonʼs correlation coefficient. c Common parental tRNAs for the top 20 ptRFs of each type showing in Venn diagrams. (TIF 535 kb) [file 13071_2019_3301_MOESM2_ESM.tif]

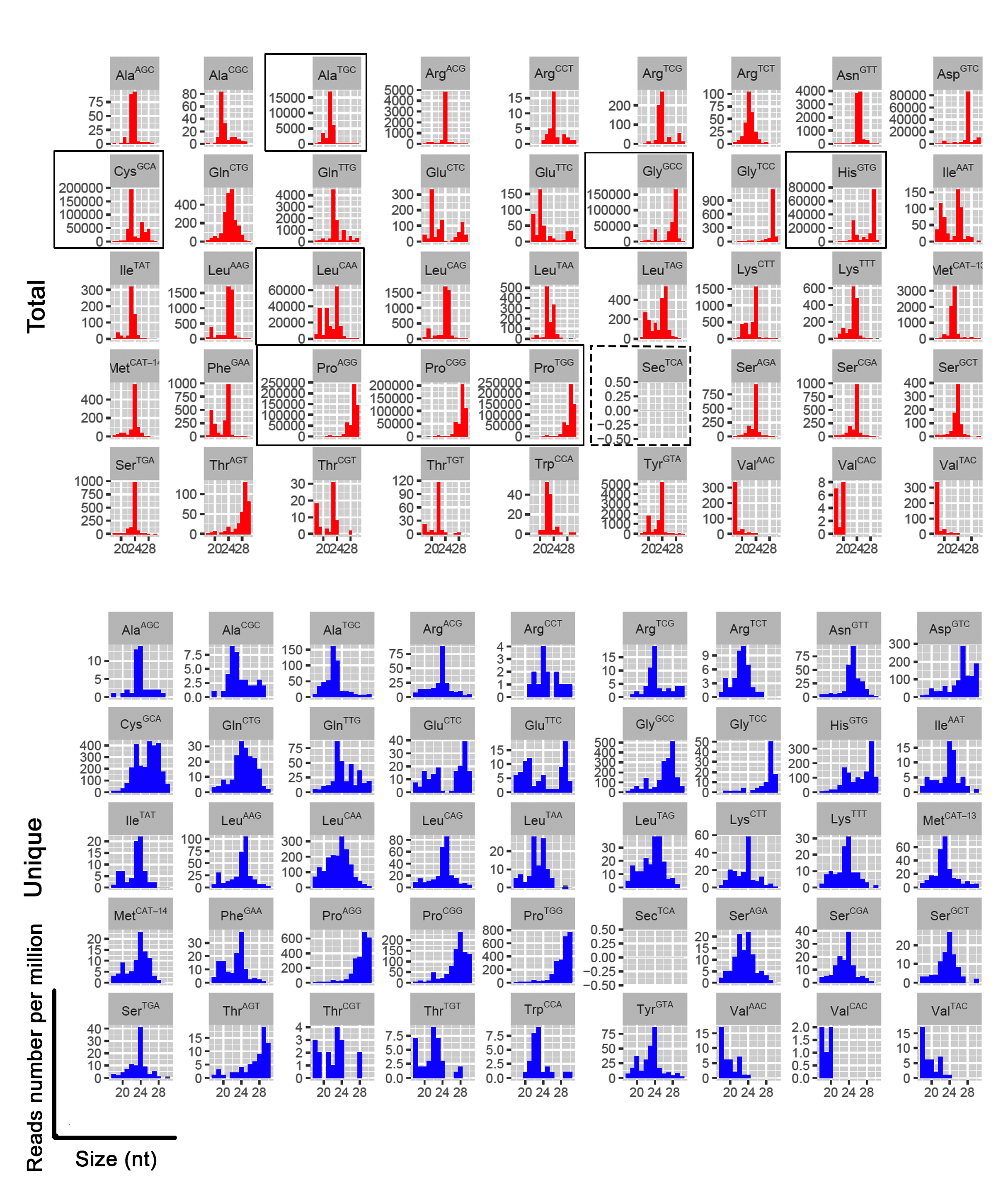

Supplement: Supplementary file 3 — Figure S2. Size distributions of the 5'ptRFs in total and unique aspects. Solid boxes represent those with RPMs above 105 and dotted boxes represent those with RPMs less than 5. (TIF 2602 kb) [file 13071_2019_3301_MOESM3_ESM.tif]

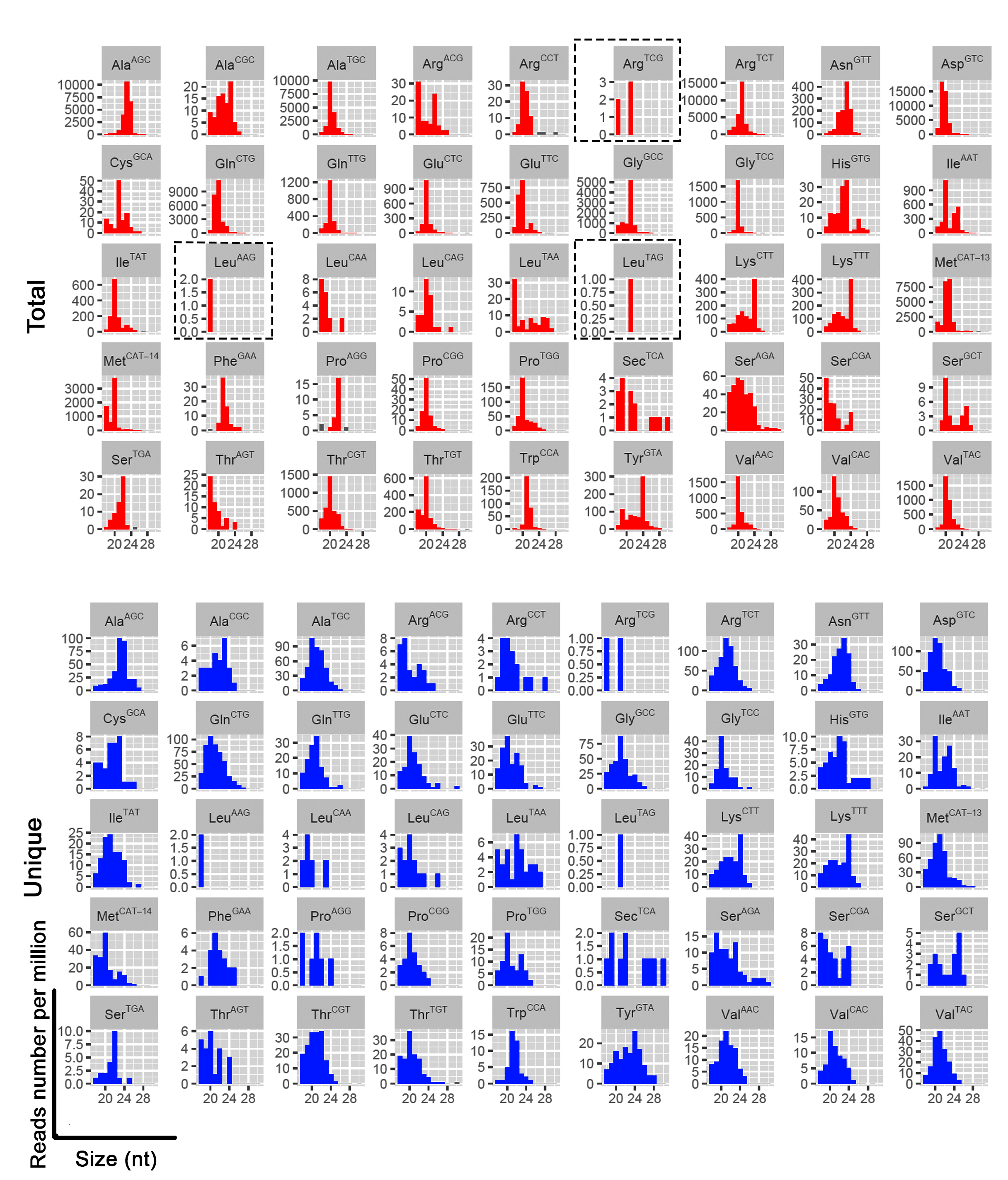

Supplement: Supplementary file 4 — Figure S3. Size distributions of the mid-ptRFs in total and unique aspects. Dotted boxes correspond to those with RPMs less than 5. (TIF 2578 kb) [file 13071_2019_3301_MOESM4_ESM.tif]

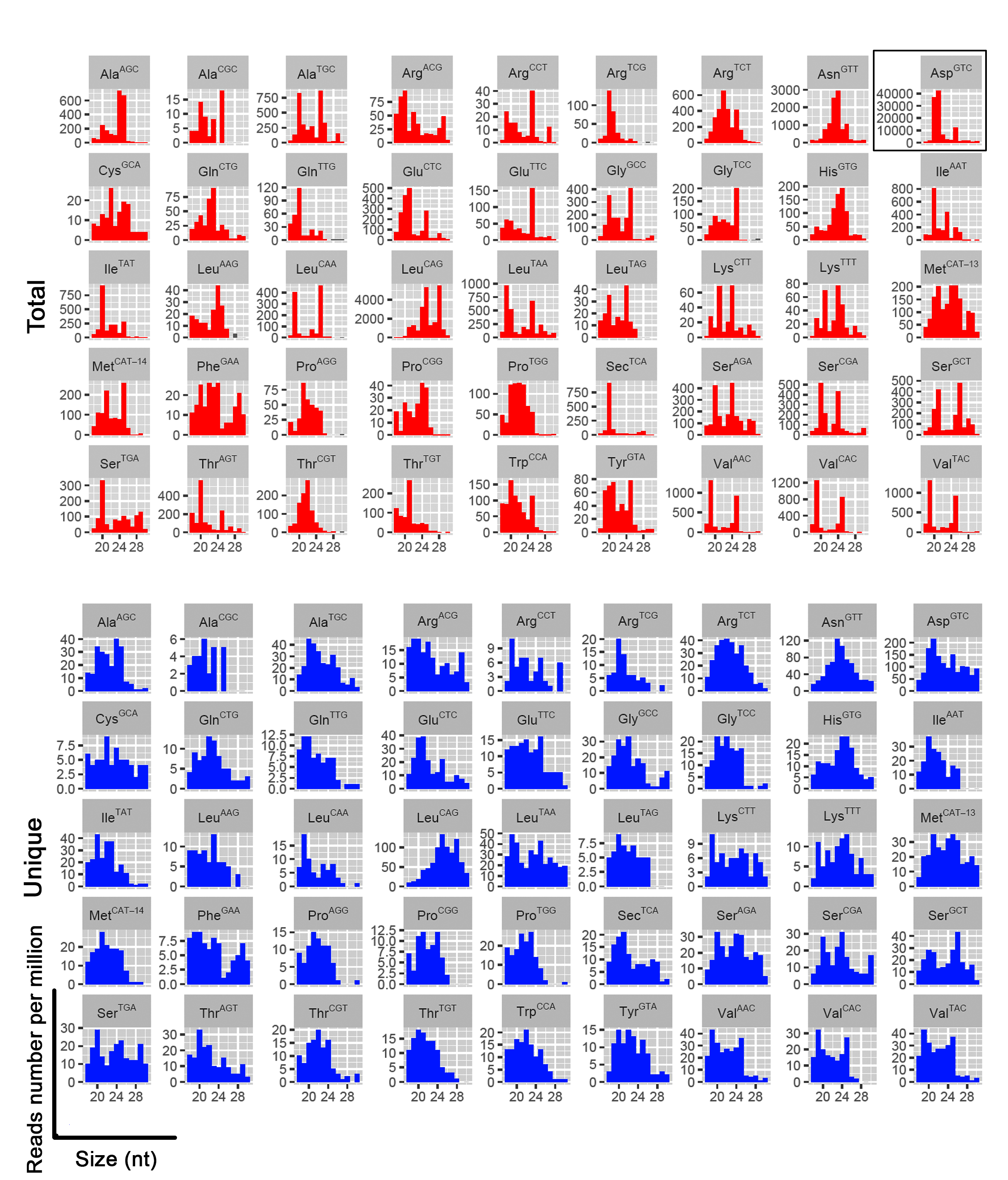

Supplement: Supplementary file 5 — Figure S4. Size distribution of the 3'ptRFs in total and unique aspects. The solid box symbolizes those with RPMs above 105. (TIF 2626 kb) [file 13071_2019_3301_MOESM5_ESM.tif]
